# Novel non-invasive particles in exhaled air method to explore the lining fluid of small airways—a European population-based cohort study

**DOI:** 10.1136/bmjresp-2020-000804

**Published:** 2021-01-05

**Authors:** Laith Hussain-Alkhateeb, Björn Bake, Mathias Holm, Össur Emilsson, Ekaterina Mirgorodskaya, Anna-Carin Olin

**Affiliations:** 1 Public Health and Community Medicine, University of Gothenburg Sahlgrenska Academy, Goteborg, Sweden; 2 Department of Respiratory Medicine and Allergology, Insitute of Medicine, Gothenburg, Sweden; 3 Department of Medical Sciences, Respiratory, allergy and sleep research, Uppsala University, Uppsala, Sweden; 4 Proteomics Core Facility, Sahlgrnska Acadey, University of Gothenburg, Gothenburg, Sweden

**Keywords:** asthma, exhaled airway markers, tobacco and the lung, non invasive ventilation

## Abstract

**Introduction:**

Respiratory tract lining fluid of small airways mainly consists of surfactant that can be investigated by collection of the particles of exhaled aerosol (PExA) method. This offers an exciting prospect to monitor small airway pathology, including subjects with asthma and smokers.

**Aim:**

To explore the influence of anthropometric factors and gender on phospholipids, surfactant protein A (SP-A) and albumin of the lining fluid of small airwaysand to examine the association with asthma and smoking. Furthermore, to examine if the surfactant components can predict lung function in terms of spirometry variables.

**Method:**

This study employs the population-based cohort of the European Community Respiratory Health Survey III, including participants from Gothenburg city, Sweden (n=200). The PExA method enabled quantitative description and analytical analysis of phospholipids, SP-A and albumin of the lining fluid of small airways.

**Results:**

Age was a significant predictor of the phospholipids. The components PC14:0/16:0, PC16:0/18:2 (PC, phosphatidylcholine) and SP-A were higher among subjects with asthma, whereas albumin was lower. Among smokers, there were higher levels particularly of di-palmitoyl-di-phosphatidyl-choline compared with non-smokers. Most phospholipids significantly predicted the spirometry variables.

**Conclusion:**

This non-invasive PExA method appears to have great potential to explore the role of lipids and proteins of surfactant in respiratory disease.

## Introduction

Monitoring inflammation in small airways (defined as airways with an inner diameter less than 2 mm) is vital during the course of treatment, for example in asthma where the inflammation of small airways is associated with increased risk for exacerbations and poorer disease control.^1^ Methods for early detection of surfactant abnormalities are scarce, however, making timely medical preventive actions difficult. Broncho-alveolar lavage (BAL) is currently the only established clinical method to retrieve biological samples from the lining fluid of small airways. Despite its widespread use, this method suffers sampling issues which may influence the sample, such as the effects of the lavage-fluid itself, contamination caused by local bleeding and an unknown dilution factor of which all can undermine the results interpretation and comparability.^2^ Another method used in research is the sampling of exhaled breath condensate. This method, however, is associated with large intra-individual variations and difficulties in the quantification due to differences in dilution and oral contamination,^3^ which hampers its interpretation.

Non-invasive methods for assessing the degree of structural changes in small airways do exist, such as measurements of inhomogeneous ventilation distribution and assessments of resistance and reactance of the respiratory system by impulse-oscillometry.^4^ However, these methods may at best reflect anatomical abnormalities of the small airways.

The sampling of lining fluid of the small airways, in the form of exhaled droplets or particles, offers a novel and exciting prospect to monitor small airway inflammation, for example in asthma and in smokers. This concept fashions the basic principle of the particles in exhaled air (PExA) method, which is described in full elsewhere.^5^ By imposing a special breathing manoeuvre to increase airway closure and reopening and sample particles in the size range of 0.4–4 µm, the PExA method enables sampling of lining fluid from small airways with acceptable reproducibility.^6^ These exhaled particles are mainly composed of surfactant, which consists of a broad range of phospholipids and proteins. Di-palmitoyl-di-phosphatidyl-choline (DPPC), the most abundant lipid, is important to keep the surface tension low. This is crucial for small airway patency, and is the result of the delicate interaction between DPPC with lung-specific proteins, for example, surfactant proteins A, B and C (SP-A, SP-B and SP-C) with SP-A being the most abundant lung-specific protein.^5^

The lining fluid phospholipids and proteins are potential biomarkers for diseases such as asthma.^7^ Furthermore, the influx of fibrinogen as well as plasma lipids through alveolar barrier may disturb surfactant function. In one of the few studies of surfactant composition in humans, the 1-palmitoyl-2-linoleoyl-*sn*-glycero-3-phosphocholine (PC 16:0/18:2) was shown to substantially increase after allergen challenge, and the increase was accompanied by an increase in the surface-tension.^8^ SP- A was explored among smokers in the context of chronic obstructive pulmonary disease in previous studies.^9 10^

While PExA method has previously been used among healthy and asthmatic subjects, assessing the influence of anthropometric factors, gender, asthma and smoking on the composition of surfactant lipids and proteins using the PExA method is novel, which was the main interest of this study. Furthermore, we analysed to what extent surfactant lipids and proteins are associated with lung function in terms of spirometry variables.

## Methods

### Patient and public involvement

Research into novel diagnostics for assessing small airway pathology is a current priority in research and clinical practices. Our non-invasive PExA method was originally designed based on data samples and feedback from patients during pilot studies in order to advance the tool acceptability and feasibility during examination and in future clinical practice.

### Selection of the study population

This study is part of a population-based cohort: The European Community Respiratory Health Survey III (ECRHS-III).^11^ Participants are randomly selected from the population of Gothenburg city, Sweden, and subjects reporting asthma symptoms enrich the sample. Only individuals who consented to more extensive examinations of the airways, using the PExA method were retained in the study. The examination took place between March 2011 and October 2012. In all, 811 subjects were invited to the clinical examination, 278 responded to the invitation and 211 subjects succeeded all examinations including the PExA method. Two hundred and seven individuals had acceptable PExA recordings without technical problems, but only 200 subjects revealed enough particle material sampled to be analysed, which ultimately defined our study population.

### Definition of healthy subjects

Subjects with lung-function measurements within normal limits (forced vital capacity (FVC), forced expiratory volume in one second (FEV_1_) and the ratio FEV_1_/FVC >0.70), who were ex-smokers or never-smokers and who did not report asthma were classified as healthy and constitute the ‘healthy’ group.

### Definitions of asthma and smoking status

All subjects filled in the ECRHS-III questionnaire, and were subsequently classified as ‘current asthma’ based on an affirmative answer to the question ‘Have you ever had asthma’, and if they were reporting of 'any attack of asthma during the past year'. Among those with current asthma (n=16), nine were ex-smokers and one current smoker. Subjects who answered that they had smoked for as long as 1 year, and ‘still smoking as of 1 month ago’, were classified as smokers. The smoking group consists of 17 current smokers, among them one subject with asthma.

### Spirometry

Spirometry was performed with an Easy One spirometer (EasyOne Plus Diagnostic, CH-8005, Zurich, Switzerland). The FVC and FEV_1_ were obtained before and after 15 min after administration of a bronchodilator, 3×0.5 mg Bricanyl (AstraZeneca, SE-151 85 Södertälje, Sweden) and the results are presented after bronchodilation. The procedures complied with international guidelines.^12^ FVC and FEV_1_ were expressed as a percentage of the reference value (% pred) according to Brisman *et al*,^13^ whereas FEV_1_/FVC is presented as a ratio.

### Exhaled particles (PExA)

The equipment for counting exhaled particles, PExA (PExA AB, Gothenburg, Sweden), has been described previously in detail.^5^ In short, a reservoir of 3.4 L is located inside a thermostatic box set at 36 °C. An optical counter (Grimm model 1.108, Grimm Aerosol Technik GmbH & Co, Ainring, Germany) draws aerosol from the reservoir at the mouth-end. At the other end of the reservoir, 36 °C fully saturated air makes up for the sampling. The Grimm counter measures particles every second and covers diameters between 0.41 and 4.55 µm. Particles were collected using a two-stage inertial impactor (PM10 Impactor; Dekati, Tampere, Finland). An ultrasonic flow metre (OEM flow sensor, Spiroson-AS, Medical Technologies, Zürich, Switzerland) measures exhalation flow and enables visualisation of the expiratory flow and volume.

The applied breathing manoeuvre has been described previously in detail^14^ and includes a full exhalation to residual volume; breath holding for 3 s, followed by a maximal inhalation to total lung capacity, immediately followed by a slow deep exhalation when the exhaled particles are being measured and sampled. Two sampling sessions were performed consecutively, each to achieve 60 L of exhaled air, after the administration of the bronchodilator. Each sampling session consisted of repeated breathing manoeuvres as described above, interrupted by short periods of tidal breathing of particle air-free until all particles in the reservoir had been counted.

The exhaled material was collected on a thin membrane of hydrophilic polytetrafluoroethylene membrane (FHLC02500, Merck Millipore Billerica, MA, USA). The membrane was divided into two and each half was transferred to a polypropylene micro tube and stored at −80°C prior to analysis. One-half of the collection membrane was used for lipid analysis by LC-MS (Liquid Chromatography-Mass Spectrometry) and the other one for protein analysis by immunoassays.

### Analysis of phospholipids

Lipids, from one half of the collection membrane, were extracted into 160 µL of methanol:chloroform:water with 40 mM ammonium acetate (6:3:2, v:v:v). For quantification, internal standards, PC17:0/14:1 and PC17:0/20:4, were added to all samples prior to extraction.

Samples were analysed in a positive ion mode on a triple quadrupole mass spectrometer (Sciex API3000, AB Sciex, Toronto, Canada) equipped with an electrospray ion source. Samples were introduced by direct flow injection using a CTC HTS PAL auto-sampler (CTC DPPC Analytics, Zwingen, Switzerland) and a Shimadzu 10ADvP LC system (Shimadzu, Kyoto, Japan) with an isocratic mobile phase containing methanol:chloroform:water with 40 mM ammonium acetate (6:3:2). Injection volume was 20 µL.

DPPC (PC 16:0/16:0) and POPC (PC 16:0/18:1) were analysed using selected reaction monitoring (SRM). SRM analysis included the transitions m/z 734.2>184.1 (DPPC), 760.2>184.1 (POPC), 718.2>184.1 (PC17:0/14:1) and 796.2>184.1 (PC17:0/20:4). Data analysis was performed with Analyst software 1.6.1. (AB Sciex, Toronto, Canada). DPPC and POPC were quantified using external calibration curves prepared with POPC and DPPC standards (Avanti Polar Lipid) in the range of 0.005–0.500 µM, corresponding to amounts of 0.6–60 ng per sample.

Precursor ion scanning of m/z 184.1 was used for detection of phosphatidylcholine-containing lipids (PC). Selected PCs were semi-quantified by comparing their extracted ion intensities with intensity of the DPPC and expressed as percent of the detected DPPC. Those were further recalculated based on the measured DPPC concentrations in each sample to obtain their weigh percent concentrations in PEx.

### Exhaled particle extraction and protein immunoassay

SP-A and albumin were analysed in extracted PEx by ELISA, and the following solvents were prepared and used for sample preparation and analysis. Extraction buffer prepared as phosphate-buffered saline (PBS) 10 mM Na Phosphate, 0.15M NaCl, containing 1% bovine serum albumin, w/v, and 0.05% Tween-20, v/v. ELISA sample diluents prepared according to ELISA manufacture recommendation. Assay buffers prepared by mixing extraction buffer and the corresponding ELISA sample diluent in the ratio 1:2, v:v.

The second half of the collection membrane was used for SP-A and albumin analyses by ELISA. Proteins were extracted from membranes using extraction buffer, prepared as PBS 10 mM Na Phosphate, 0.15M NaCl, containing 1% bovine serum albumin, w/v, and 0.05% Tween-20, v/v. To each sample, 140 µL of the extraction buffer was added, followed up by 60 min shaking at 400 rpm and 37°C in a thermomixer (Thermomixer comfort, Eppendorf; Eppendorf AG, Hamburg, Germany). Three polypropylene vials, each containing 40 µL of extract, were prepared and stored at −20°C prior to further analysis. One vial was used for SP-A assay, another for the albumin assay and the third vial was maintained as reserve sample.

Prior to immunoassays, samples were thawed to room temperature and diluted three times with provided ELISA sample diluents. The assays were performed according to the manufacturer’s instructions, with minor modifications to the buffer composition and incubation time. All calibrants and controls were prepared and assayed in the same assay buffer as particle samples. The absorbance at 450 nm was measured using a plate-reader from BioTek ELx-808UI (Highland Park, MI, USA).

SP-A in extracted particle samples was quantified using a human SP-A ELISA kit (RD191139200R, BioVendor, Czech Republic). The plate incubation time was extended to 3 hours. Under these experimental conditions, the limit of quantification (LOQ) for SP-A detection was 0.5 ngμmL^−1^ as determined by precision profile at 15% coefficient of variation (CV).

Albumin was quantified using human albumin ELISA kit (E-80AL, Immunology Consultants Laboratory, USA) The plate incubation time was extended to 1.5 hours. The LOQ for albumin was 0.9 ngμmL^−1^ as determined by precision profile at 15% CV.

### Calculation of mass and concentrations

The total number of exhaled particles was calculated as a sum of particles detected in both sessions. The exhaled particle mass was calculated based on the number of exhaled particles, assuming the particles are of spherical shape and have a water-identical density. The obtained particle data are presented as number concentrations and expressed per litre of exhaled breath—PEx number concentration expressed as n×1000 (kn) per litre of exhaled breath, kn/L.

Average results of the two sampling sessions are computed. SP-A, albumin and lipids concentration in collected samples were expressed as a weigh percent (wt%), obtained by dividing the quantified protein or lipid mass by the total particle mass and expressed as a percentage, whereas PC14:0/16:0 and PC16:0/18:2 are expressed as a percentage or the DPPC concentration.

### Statistical analysis

Descriptive statistics of the mean and SD were sought for describing the individuals’ background and characteristics as well as their phospholipids and proteins profile across ‘healthy’, ‘asthma’ and ‘smokers’ groups.

Although our data have generally satisfied the linearity assumptions, the quantile regression method was employed for assessing the associations of lipids and proteins by the surfactants and lung functions. Quantile regression is robust for handling extreme observations and skewed data if it exists, and superiorly able to derive a more detailed picture on the relationship between the outcomes and our list of predictors, at different percentile levels.^15^ Following several attempts of exploring the associations of lipids and proteins by lung functions of different percentile levels, the 50th percentile was eventually presented as most appropriate for the data. While considering the relatively small study population with an extended list of a potentially contributing factors in the pathways of these assessments, we based our list of covariates in the model on a priori biological assumptions.

Best adjusted model was retained using the '*link test'* method which assumes that if a regression equation is properly specified, we should be able to find no additional independent variables that are significant except by chance.^16^ Different models contained different confounders but generally, those were of characteristics (age, sex), anthropometric (weight, height or BMI) or other factors like atopy, which is the genetic tendency of developing allergic diseases such as asthma, and PEx concentration. For all statistical analyses, p-values of <0.05 were considered statistically significant.

For the statistical analyses, STATA V.15.1 (*Stata Statistical Software: Release 15*) was used.

## Results

Table 1 summarises the general characteristics of the subjects. The mean age of participants was similar across all groups with an average of 53 years, but sex was slightly different across the groups, with females being more prevalent in the asthmatic group. Spirometry results showed an obstructive pattern among subjects with asthma.

**Table 1:** Description of background, characteristics and lung functions across healthy, asthmatic and smokers. mean and standard deviation (SD)

Background and characteristic	Healthy (n=168)		Asthma (n=16)		Smokers (n=17)		All (n=200)	
	Mean	SD	Mean	SD	Mean	SD	Mean	SD
Age (year)	53.1	7.3	53.5	7.2	55.1	5.1	53.2	7.2
Sex, female (%)	51.2	–	75.0	–	58.8	–	53.5	–
BMI (kg/m^2^)	26.8	3.6	28.4	5.5	26.7	3.0	26.9	3.7
FEV_1_ (% pred)	95.1	12.9	85.2	13.7	94.7	11.0	94.4	13.4
FVC (% pred)	96.2	12.0	93.0	10.7	98.3	10.0	96.2	11.9
FEV_1_/FVC	0.78	0.05	0.72	0.08	0.76	0.06	0.77	0.05

BMI, body mass index; FEV_1_, forced expiratory volume in one second; FVC, forced vital capacity.

The concentrations of lipids and proteins are displayed in table 2. Among the lipids, DPPC was highest among smokers. POPC, PC14:0/16:0 and PC16:0/18:2 revealed no obvious differences among the groups and there were no evident differences across the groups with respect to the measured proteins.

**Table 2:** Description of the average concentrations of phospholipids and proteins across healthy, subjects with asthma and smoker groups, ECRHS-III (n=200)

Phospholipids and proteins concentration	Healthy (n=168)		Asthma (n=16)		Smokers (n=17)		Total (n=200)	
	Mean	SD	Mean	SD	Mean	SD	Mean	SD
DPPC (wt%)	11.9	2.6	11.3	2.2	13.7	3.8	11.9	2.5
POPC (wt%)	3.0	0.8	2.9	0.8	3.5	1.0	3.0	0.7
PC14:0/16:0 (%)	21.5	5.1	23.2	4.3	23.0	5.4	21.5	4.9
PC16:0/18:2 (%)	9.9	2.2	11.1	1.3	9.6	1.9	10.0	2.1
SP-A (wt%)	3.3	1.1	3.4	1.5	3.7	0.9	3.3	1.1
Albumin (wt%)	6.8	2.7	5.6	2.1	5.7	1.3	6.7	2.7
PEx (kn/L)	7.6	6.9	6.6	5.8	9.8	6.9	7.5	6.7

DPPC, di-palmitoyl-di-phosphatidyl-choline; ECRHS-III, European Community Respiratory Health Survey III; PC, phosphatidylcholine; PEx, particles of exhale; SP-A, surfactant protein A.

All lipids were significantly associated with age but the direction differed, as presented in table 3. DPPC and POPC decreased with age whereas PC14:0*/16:0 and PC16:0/18:2* increased. Among the proteins, albumin increased significantly with increasing age. Sex was not a significant predictor of any of the lipids or proteins, but weight and height significantly predicted *PC14:0/16:0* only. Asthma was significantly associated with an increase of *PC14:0/16:0, PC16:0/18:2* and SP-A, but a decrease of albumin. Current smoking was associated with increased levels of DPPC, POPC and SP-A.

**Table 3:** Adjusted quantile regression (50th) analysis of surfactant composition (*phospholipids and proteins*) by the background characteristics, asthma and smoking (n=200)

Explanatory variables	DPPC (wt%) coefficient (95% CI)	POPC (wt%) coefficient (95% CI)	PC14:0/16:0 (%) coefficient (95% CI)	PC16:0/18:2 (%) coefficient (95% CI)	SP-A (wt%) coefficient (95% CI)	Albumin (wt%) coefficient (95% CI)
Age (years)	−0.07 (−0.14 to −0.01)*	−0.03 (−0.04 to −0.01)*	0.14 (0.01 to 0.26)*	0.07 (0.01 to 0.12)*	−0.01 (−0.04 to 0.02)	0.07 (0.02 to 0.11)*
Sex, male (ref)	0.60 (−0.10 to 1.30)	0.06 (−0.20 to 0.32)	−0.88 (−2.87 to 1.12)	−0.46 (−1.44 to 0.52)	−0.15 (−0.57 to 0.27)	−0.40 (−1.34 to 0.54)
Weight (kg)	0.01 (−0.03 to 0.04)	−0.01 (−0.10 to 0.10)	0.12 (0.01 to 0.25)*	0.01 (−0.03 to 0.05)	−0.02 (−0.03 to 0.01)	−0.02 (−0.04 to 0.01)
Height (cm)	0.01 (−0.05 to 0.07)	0.01 (−0.01 to 0.03)	0.14 (0.01 to 0.26)*	0.02 (−0.04 to 0.08)	−0.01 (−0.03 to 0.03)	−0.03 (−0.08 to 0.01)
BMI (kg/m^2^)	0.55 (−0.05 to 1.15)	0.19 (−0.10 to 0.39)	−0.77 (−2.02 to 0.47)	−0.08 (−0.78 to 0.62)	−0.22 (−0.51 to 0.08)	0.10 (−0.42 to 0.62)
Asthma	−1.11 (−2.81 to 0.57)	−0.19 (−0.75 to 0.37)	3.43 (0.05 to 6.93)*	1.75 (0.09 to 3.41)*	0.71 (0.03 to 1.39)*	−1.21 (−2.33 to −0.10)*
Smokers	1.82 (0.50 to 3.14)*	0.41 (0.04 to 0.80)*	1.50 (−1.33 to 4.33)	0.18 (−1.25 to 1.60)	0.80 (0.11 to 1.49)*	−0.42 (−1.56 to 0.71)

Significant associations (*) are also marked in bold.

BMI, body mass index; DPPC, di-palmitoyl-di-phosphatidyl-choline; ECRHS-III, European Community Respiratory Health Survey III; PC, phosphatidylcholine; SP-A, surfactant protein A.

Many of the phospholipids were significantly associated with the lung-function variables as illustrated in table 4. FVC and FEV_1_/FVC were significantly associated with DPPC, and both increased as DPPC increased. POPC was also significantly associated with both FVC and FEV_1_/FVC. PC14:0/16:0 was significantly associated with FVC and FEV_1_ although showing different directions of associations. PC16:0/18:2 was not associated with the spirometry variables.

**Table 4:** Crude and adjusted quantile regression (50th) analysis of lung function by surfactant composition (*lipids and proteins*) among healthy groups, ECRHS-III (n=200)

Explanatory variables	FEV_1_ (%pred) coefficient (95% CI)		FVC (%pred) coefficient (95% CI)		FEV_1_/FVC coefficient (95% CI)	
	Crude	Adjusted	Crude	Adjusted	Crude	Adjusted
DPPC (wt%)	−0.01 (−0.08 to 0.06)	−0.03 (−0.10 to 0.04)	−0.03 (−0.11 to 0.06)	0.03 (0.01 to 0.06)*	0.16 (0.002 to 0.31)*	0.25 (0.10 to 0.40)*
POPC (wt%)	−0.02 (−0.24 to 0.20)	−0.12 (−0.36 to 0.11)	−0.09 (−0.37 to 0.20)	0.14 (0.06 to 0.22)*	0.45 (0.06 to 0.95)*	0.91 (0.42 to 1.42)*
PC14:0/16:0 (%)	0.02 (−0.01 to 0.05)	−0.02 (0.001 to 0.03)*	0.04 (0.001 to 0.08)*	0.02 (0.001 to 0.04)*	−0.08 (−0.15 to −0.01)*	−0.04 (−0.08 to 0.001)
PC16:0/18:2 (%)	0.01 (−0.06 to 0.08)	0.03 (−0.05 to 0.10)	0.01 (−0.08 to 0.11)	0.04 (−0.06 to 0.13)	−0.10 (−0.25 to 0.07)	−0.01 (−0.10 to 0.08)
SP-A (wt%)	0.03 (−0.10 to 0.15)	0.01 (−0.06 to 0.07)	0.09 (−0.08 to 0.25)	0.13 (−0.06 to 0.33)	0.09 (−0.20 to 0.37)	0.04 (−0.06 to 0.13)
Albumin (wt%)	−0.02 (−0.07 to 0.02)	−0.02 (−0.05 to 0.01)	−0.03 (−0.10 to 0.03)	−0.03 (−0.10 to 0.03)	−0.06 (−0.19 to 0.06)	−0.04 (−0.04 to 0.08)

Significant associations (*) are also marked in bold.

DPPC, di-palmitoyl-di-phosphatidyl-choline; ECRHS-III, European Community Respiratory Health Survey III; FEV_1_, forced expiratory volume in one second; FVC, forced vital capacity; PC, phosphatidylcholine; SP-A, surfactant protein A.

None of the proteins (SP-A and albumin) showed any significant association with any of the lung function variables.

## Discussion

This is the first population-based study to explore surfactant phospholipids and proteins in the lining fluid of small airways, using the novel non-invasive method, PExA. The distribution of phospholipids and proteins of surfactant (DPPC, POPC, SP-A and albumin) were determined quantitatively and expressed as wt% of the sample mass, whereas PC14:0/16:0 and PC16:0/18:2 are expressed relative to the DPPC concentration.

The variation in concentrations of the major components of the surfactant varied in general within healthy individuals, and were only to small degree influenced by height, weight and sex in healthy individuals, which might be expected. One of the major functions of surfactant—primarily to reduce surface tension—is highly dependent on the preservation of its complex composition, where DPPC is of highest importance to reduce surface tension to keep small airways open and enhances oxygen transfer.^17^

Age influenced almost all measured components of the surfactant; however, the cause of the decrease of both DPPC and POPC and the increase in albumin with age remains unknown. Physiological measurements, for instance FEV_1_ and FVC, decrease with age but this is likely due to decreased elasticity of the lung parenchyma. Airway closure and reopening increase with increasing age^18 19^ and it is conceivable that the changes in surfactant phospholipids and proteins increase surface tension in small airways as to increase airway closure and opening. Exhaled concentration of particles increases with age,^20^ in agreement with increasing airway closure and opening.

DPPC was increased in smokers, and the increase was substantial—1.82 (0.50–3.14) wt% higher in smokers, which is on average approximately 15% higher than in non-smokers. Growing evidence suggests that dysfunctional lipid metabolism plays an important role in the development of respiratory disease and reflects ongoing inflammatory processes.^21^ In theory, smoking-induced DPPC increase could result in surfactant dysfunction and increased airway closure and increase exhaled particle concentration, which is in line with the present findings.

A study by Wright *et al* revealed that lower levels of DPPC were observed in BAL with a decreasing trend among more severe asthma subjects.^22^ In the present study, while the DPPC levels were reduced in asthma (Coef −1.11 wt%, CI: −2.81 to 0.57, compared with healthy subjects), the non-significant results were probably caused by small sample size and, subjects with rather mild and well-controlled asthma. The largest difference between asthma and healthy subjects wasin the phospholipid PC16:0/14:0, which warrant further assessment in larger studies. In adjusted models, we also found increased levels of SP-A in PEx in asthma.

The PExA non-invasive approach managed to quantify, or semi-quantify, five different phospholipid species, all determined in positive mode. This has specifically excluded the phosphatidyl-glycerols, which is nevertheless only possible to detect in negative mode— an acknowledged limitation in this method. Moreover, other low abundant phospholipids, like ethanol-amines and ceramides, were not possible to quantify with the present mass-spectrometer. Another limitation related to the design of the study is the low number of subjects with asthma and current smokers. This hinders early drawing of any conclusions based on the derived findings, although interesting alterations of patterns of the major phospholipids in both smokers and subjects with asthma were observed, which warrant larger studies dedicated to that purpose. In addition, the classification of asthma was based on self-reported asthma in combination with the reporting of an asthma attack within the last year. This may introduce a non-differential misclassification of disease which tends to bias the outcomes towards the null, that is, reduce the strength of the association between asthma and the surfactant composition.

Novel and highly sensitive analytical techniques are promising and can permit a much larger set of lipids including cholesterol, which is of importance to the surfactant structure.^23^ In this population-based sample, subjects with asthma were well controlled at examinations and had mild-moderate disease severity, most probably with limited airway inflammation in the small airways. Subjects with severe asthma and severe small airway disease may show more deviant surfactant components.

The sampling method proposed by PExA is applicable to study surfactant biology in larger groups, non-invasively. This is a great advantage over other available methods and findings from the PExA method appear to have great potential enabling studies of the role of phospholipids and proteins of the surfactant in respiratory diseases.
